# The Irreversible Loss of a Decomposition Pathway Marks the Single Origin of an Ectomycorrhizal Symbiosis

**DOI:** 10.1371/journal.pone.0039597

**Published:** 2012-07-18

**Authors:** Benjamin E. Wolfe, Rodham E. Tulloss, Anne Pringle

**Affiliations:** 1 FAS Center for Systems Biology, Harvard University, Cambridge, Massachusetts, United States of America; 2 Herbarium Rooseveltensis Amanitarum, Roosevelt, New Jersey, United States of America; 3 Honorary Research Associate, the New York Botanical Garden, Bronx, New York, United States of America; 4 Department of Organismic and Evolutionary Biology, Harvard University, Cambridge, Massachusetts, United States of America; Duke University Medical Center, United States of America

## Abstract

Microbial symbioses have evolved repeatedly across the tree of life, but the genetic changes underlying transitions to symbiosis are largely unknown, especially for eukaryotic microbial symbionts. We used the genus *Amanita,* an iconic group of mushroom-forming fungi engaged in ectomycorrhizal symbioses with plants, to identify both the origins and potential genetic changes maintaining the stability of this mutualism. A multi-gene phylogeny reveals one origin of the symbiosis within *Amanit*a, with a single transition from saprotrophic decomposition of dead organic matter to biotrophic dependence on host plants for carbon. Associated with this transition are the losses of two cellulase genes, each of which plays a critical role in extracellular decomposition of organic matter. However a third gene, which acts at later stages in cellulose decomposition, is retained by many, but not all, ectomycorrhizal species. Experiments confirm that symbiotic *Amanita* species have lost the ability to grow on complex organic matter and have therefore lost the capacity to live in forest soils without carbon supplied by a host plant. Irreversible losses of decomposition pathways are likely to play key roles in the evolutionary stability of these ubiquitous mutualisms.

## Introduction

Diverse prokaryotic and eukaryotic microbes have evolved to form mutualistic symbioses with eukaryotic hosts, conjoining the evolutionary trajectories of distant lineages on the tree of life [1], [2]. While the diversity and function of these microbial mutualisms is increasingly apparent [3], [4], [5], [6], [7], the evolutionary origins of most microbial symbioses remain enigmatic. It is often unclear if microbial mutualists evolved from free-living relatives or parasites, or how frequently mutualisms break down and revert to autonomous or parasitic lifestyles [8], [9]. While theory predicts which conditions should favor the persistence of mutualism [10], [11], genetic mechanisms explaining the stability of real world mutualisms are generally lacking.

Comparisons between endosymbiotic and free-living Proteobacteria have identified a number of genetic changes associated with the transition to mutualism, including the loss of genes needed for autonomous growth [1], [4], [12]. However, it is unclear if patterns associated with the evolution of prokaryotic endosymbioses can be generalized to other phylogenetically and functionally distinct symbioses, including mutualisms of ectosymbionts or eukaryotic microbes. Another obstacle to understanding the origins of symbiosis is the limited number of biological systems with tractable, clearly defined, and closely related symbiotic and free-living species. While major transitions between free-living and symbiotic niches have been identified across large-scale phylogenies [9], [13], the fine-scale mapping of these transitions is hampered by poorly resolved phylogenies, and experiments are made difficult by our limited ability to manipulate free-living and symbiotic taxa [8].

Ectomycorrhizal (EM) symbioses between fungi and plants are found in many forests throughout the world and generally function as mutualisms [14]. Ectomycorrhizal fungi obtain carbon from plants in the form of simple sugars and in return, provide nutrients scavenged from the soil [15]. The symbiotic interface of the association is the ectomycorrhizal root tip; EM fungi colonize the root surface and grow between (but do not penetrate) plant cells. At coarse phylogenetic scales in the Agaricales (the gilled mushrooms), the EM symbiosis appears to have evolved independently at least 11 times, and always from autonomous, saprotrophic (SAP) fungi [13]. At finer phylogenetic scales, the lability of the symbiosis is unknown.

Whether or not EM fungi retain some capacity for autonomous growth is also unclear, and may be a key to understanding the lability of the symbiosis. A hallmark of SAP fungi is the ability to produce significant quantities of cellulases and other glycoside hydrolases, which can degrade plant cell walls to sugars [16]. Because EM fungi appear to have evolved from SAP species, they may retain genes encoding these enzymes and also degrade complex organic matter in soils. However, most of the carbon in the biomass of EM fungi appears to be derived from recently-fixed carbon [17], [18], [19], suggesting that most carbon is taken from living plant hosts or from recently deposited litter. In contrast, experiments have shown that some EM fungi may secrete cellulases [20] and the ecological significance of these data is intensely debated [21], [22]. Genome sequences of the EM fungi *Laccaria bicolor* and *Tuber melanosporum* demonstrate that some glycoside hydrolases involved in the decomposition of cellulose are retained and expressed by EM fungi, while others are lost or reduced in number [23], [24], [25]. But because there are no data for closely related free-living and symbiotic species, patterns of gene loss cannot be directly linked to the evolution of EM symbioses. If symbiotic fungi have lost the capacity for autonomous growth, transitions away from symbiosis may be unlikely.

**Figure 1:**
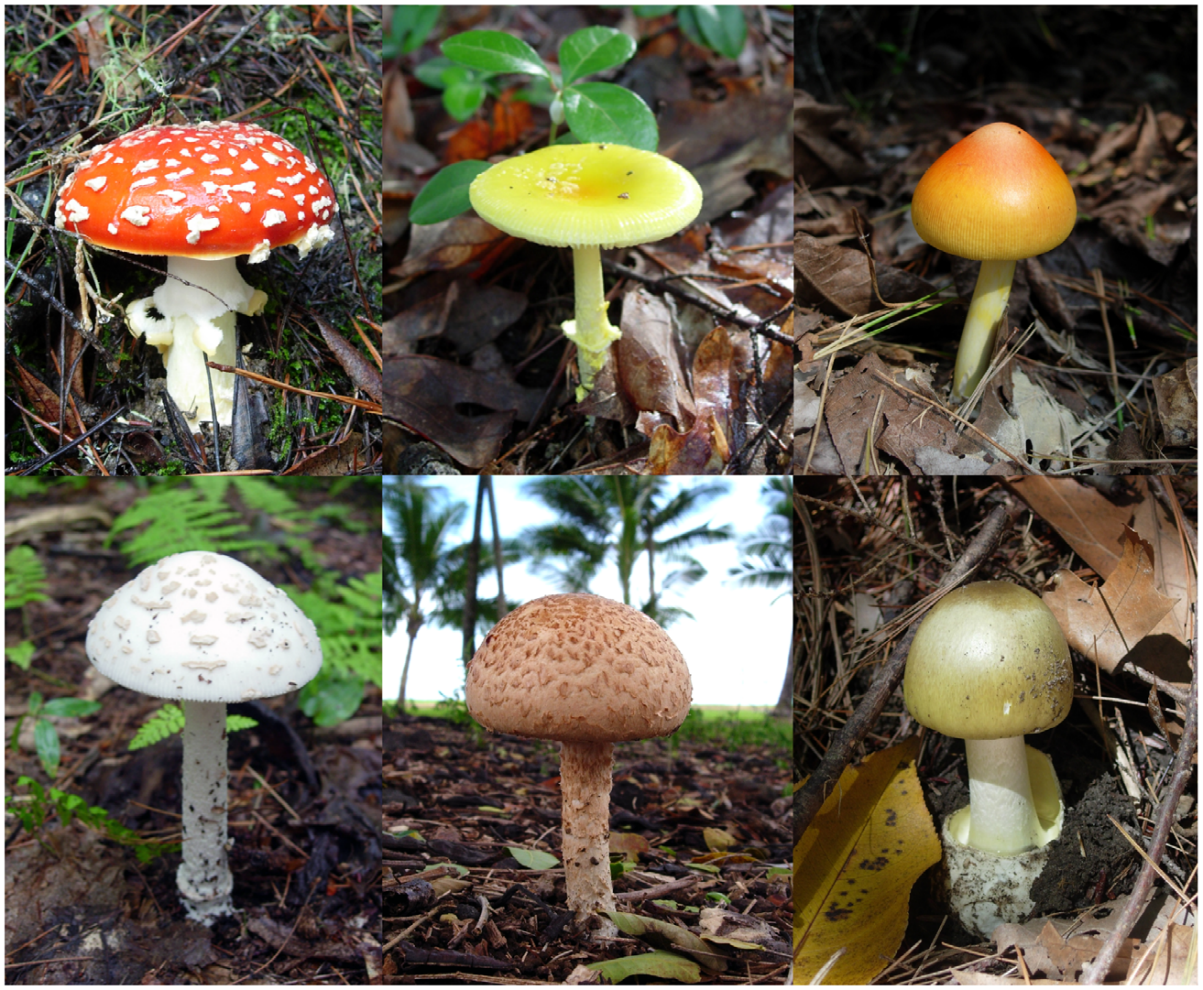
A sampling of the diversity of color and form within the genus *Amanita*. Clockwise, from top left, *Amanita muscaria* subsp. *flavivolvata*, *Amanita frostiana*, *Amanita jacksonii*, an undescribed *Amanita* species, the saprotrophic *Amanita manicata*, and *Amanita phalloides*.

By focusing on a single, diverse clade of both EM and SAP fungi, we sought to determine the origins of an EM symbiosis at fine phylogenetic scales and track the fate of genes involved in saprotrophy. The genus *Amanita* encompasses over 500 species and is found on all continents except Antarctica [26], [27], [28]. It is one of the most widely recognized groups of mushroom-forming fungi in the world [29] (Figure 1). Ectomycorrhizal *Amanita* species generally function as mutualists, and diverse host plant species show increased nutrient uptake and growth when inoculated with *Amanita* [30], [31], [32], [33], [34]. Although most species of *Amanita* are symbiotic, a small number of species consistently grow apart from woody plant hosts as free-living saprobes [35], [36]. The existence of closely related symbiotic and free-living species makes *Amanita* an ideal system for exploring the origins of mutualism and mechanisms maintaining it.

Using a multi-gene phylogenetic reconstruction of symbiotic (EM) and free-living (SAP) *Amanita* species, we determined the number of origins of the EM symbiosis within the genus. We also tracked the fate of genes in the cellulose degradation pathway, a major pathway used by free-living fungi to obtain carbon [16]. Because EM fungi use living plants as a major carbon source, we hypothesized that EM *Amanita* species would have lost genes that play essential roles in the cellulose degradation pathway. We also hypothesized that EM *Amanita* species would be unable to degrade complex carbon and grow as decomposers. By placing our functional gene and experimental data in a phylogenetic context, we highlight the potential role of irreversible gene loss in maintaining the evolutionary stability of this common mutualism.

## Results and Discussion

### Evolutionary Origin of Symbiosis within *Amanita*

**Figure 2:**
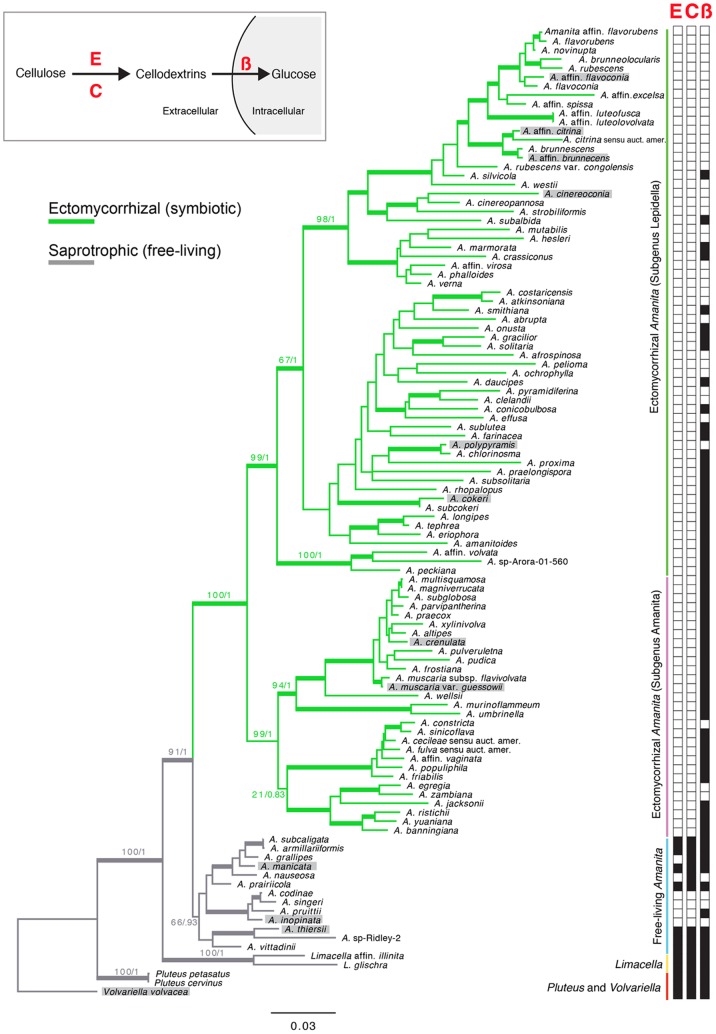
Maximum likelihood phylogeny of *Amanita* and closely related taxa. Support values at nodes indicate maximum likelihood bootstrap (MLB)/Bayesian posterior probability (BPP) values. Thick branches indicate branches with >90% MLB and >0.95 BPP. Boxes along right indicate presence/absence of three cellulase genes: endoglucanase (E), cellobiohydrolase (C), and beta-glucosidase (ß). Black  =  present. White  =  absent. Species highlighted in grey are kept as cultures and were used for experimental assessment of saprotrophy (see text). Inset indicates putative role and location of cellulases in cellulose degradation pathway. Colors of branches are based on parsimony reconstructions of trophic status.

A phylogenetic reconstruction of *Amanita* provides evidence for a single origin of the EM symbiosis (Figure 2). One monophyletic clade encompasses all obligately EM species, while free-living *Amanita* species form a second clade near the SAP genera *Volvariella*, *Pluteus* and *Limacella*. Ancestral state reconstruction supports a single origin of symbiosis in *Amanita*, and a high proportional likelihood (0.997) suggests the most recent common ancestor of the monophyletic clade of ectomycorrhizal *Amanita* species was ectomycorrhizal (Figure 2). The reconstruction of the most recent common ancestor of all *Amanita* species was ambiguous, while the most recent common ancestor of the clade encompassing *Amanita* and *Limacella* was reconstructed as saprotrophic.

### Fate of Genes Involved in the Cellulose Degradation Pathway

Saprotrophic fungi require three classes of enzymes to efficiently transform the cellulose in dead plant material into simple sugars: endoglucanases, cellobiohydrolases, and beta-glucosidases [16] (Figure 2). We targeted specific homologs of these cellulases known to play important roles in the decomposition of cellulose by *V. volvacea* [37], [38], [39], [40], a SAP species closely related to *Amanita*. The *V. volvacea* endoglucanase, encoded by *eg1*, belongs to the carbohydrate-active enzymes (CAZymes) glycoside hydrolase (GH) family 5, and is extracellular [38]. The *V. volvacea* cellobiohydrolase, encoded by *cbhI-I*, is a GH7, and is also extracellular [37]. The *V. volvacea* beta-glucosidase, encoded by *bgl*, is a GH3, and is intracellular [40]. While other CAZymes can play important roles in the decomposition of cellulose [16], most of the previous work on cellulose decomposition has focused on these three enzymes, making them a logical target for our own research.

The evolution of symbiosis in *Amanita* is associated with the loss of two of these three classes of cellulase genes. We were unable to detect copies of an endoglucanase (*eg1*) or a cellobiohydrolase (*cbhI-I*) from any EM *Amanita* species, but many SAP *Amanita* species have copies of one or both of these genes (Figure 2, S1, and S2). Southern blots confirm that lack of gene detection using PCR screens corresponds to the loss of *eg1* and *cbhI-I* from genomes, and is not caused by problems with primer design or other PCR artifacts (Figure S3).

**Figure 3:**
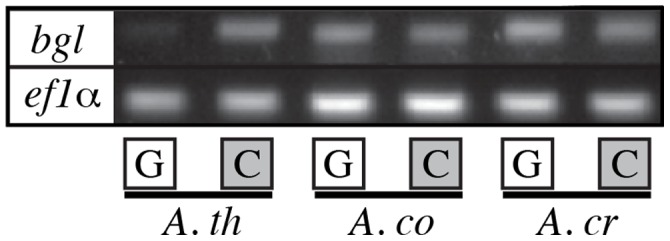
Transcript abundance of a beta-glucosidase (*bgl)* and elongation factor 1-alpha *(ef1-α)* for three species of *Amanita* growing on glucose (G) or cellobiose (C). *A. th  =  Amanita thiersii* (saprotrophic). *A. co  =  Amanita cokeri* (ectomycorrhizal). *A. cr  =  Amanita crenulata* (ectomycorrhizal).

In contrast to the losses of endoglucanases and cellobiohydrolases, homologs of a beta-glucosidase (*bgl*) were detected in both SAP and EM species (Figs. 2; S4). The presence of *bgl* was not consistent across the phylogeny; many species in subgenus *Lepidella* have lost *bgl* while most species in subgenus *Amanita* have retained *bgl* (Figure 2). Results from RT-PCR with three *Amanita* species suggest that the *bgl* genes in EM *Amanita* are still expressed (Figure 3), but the patterns of expression in response to carbon availability appear to have shifted following the evolution of the EM symbiosis.

While most free-living *Amanita* and outgroup species house genes encoding all three cellulase enzymes, species in one group of free-living *Amanita* species (encompassing *A. codinae*, *A. singeri*, *A. pruittii* and *A. inopinata*, Figure 2) lacked all three genes, with the exception of *A. pruittii*, which retained a copy of *bgl*. These fungi are not collected in association with ectomycorrhizal hosts [41], [42] and we failed to find evidence of symbiotic root structures when we searched for them in a natural population of *A. inopinata*. Inoculation of uncolonized *Pinus* seedlings with *A. inopinata* in the laboratory did not result in any sign of a mutualistic or pathogenic interaction with plants, while an ectomycorrhizal control species (*A. muscaria*) in the same experiment formed obvious EM root tips (Figure S5). These *Amanita* species may possess other types of cellulases not targeted by our study.

Endoglucanases and cellobiohydrolases are important in the initial stages of decomposition, and break down large polymers of cellulose, while beta–glucosidases convert short cellulose molecules, such as cellobiose, into glucose during the final stages of cellulose decomposition [16]. The retention and expression of *bgl* in many EM *Amanita* suggests these species have retained a limited capacity to obtain carbon from the hydrolysis of end products of cellulose degradation. However, these EM *Amanita* would rely on co-occurring fungi and bacteria to make cellobiose available as a carbon source.

The loss of endoglucanases in EM *Amanita* species contrasts with the presence and functional significance of endoglucanases in other EM species. Both the *T. melanosporum* and *L. bicolor* genomes house endoglucanases closely related to those identified in free-living, saprotrophic *Amanita* species (Figure S1). In the symbiotic root tips of *T. melanosporum*, this endoglucanase is highly expressed, suggesting it has a role in the function of the EM symbiosis. The retention of endoglucanases by some EM fungi, but loss in symbiotic *Amanita,* confirms Martin *et al.*’s hypothesis [25] of different ‘molecular tool kits’ used by different species to form symbioses.

All EM *Amanita* species have lost the *cbhI-I* gene, a CBHI cellobiohydrolase. The loss of CBHI cellobiohydrolases was also observed from the genome sequences of *T. melanosporum* and *L. bicolor* [23], [25]. To test whether loss of GH7 cellobiohydrolases is consistent across other EM fungal lineages, we conducted a PCR screen of species in sister EM-SAP clades at two other independent origins of the EM symbiosis: *Tricholoma* (EM) *vs. Clitocybe*/*Lepista* (SAP) and *Hebeloma* (EM) *vs. Galerina*/*Psilocybe* (SAP) [13]. These PCR screens confirmed that CBHI cellobiohydrolases are absent from most EM genomes, but are present in all SAP genomes (Figure S6). Studies that have cloned and sequenced CBHI cellobiohydrolases from fungal communities in a variety of forest ecosystems have not yet matched a CBHI to an EM fungal clade [43], [44], providing additional support for the loss of CBHI cellobiohydrolases as a consistent genomic hallmark of the EM symbiosis.

### Experimental Evidence for Loss of Saprotrophy

The loss of two key genes in the cellulose degradation pathway suggests EM *Amanita* can no longer function as saprotrophs. However, EM *Amanita* may possess other classes of cellulase genes or alternative decomposition pathways that could unlock available carbon from organic matter in forest soils. To test if the loss of cellulases correlates with the loss of the capacity to grow on complex organic matter, we grew axenic cultures of nine EM *Amanita*, as well as three free-living *Amanita* species and two outgroup species (Table S3) in media where sterile litter was the sole source of carbon. Target species span our phylogeny.

**Figure 4:**
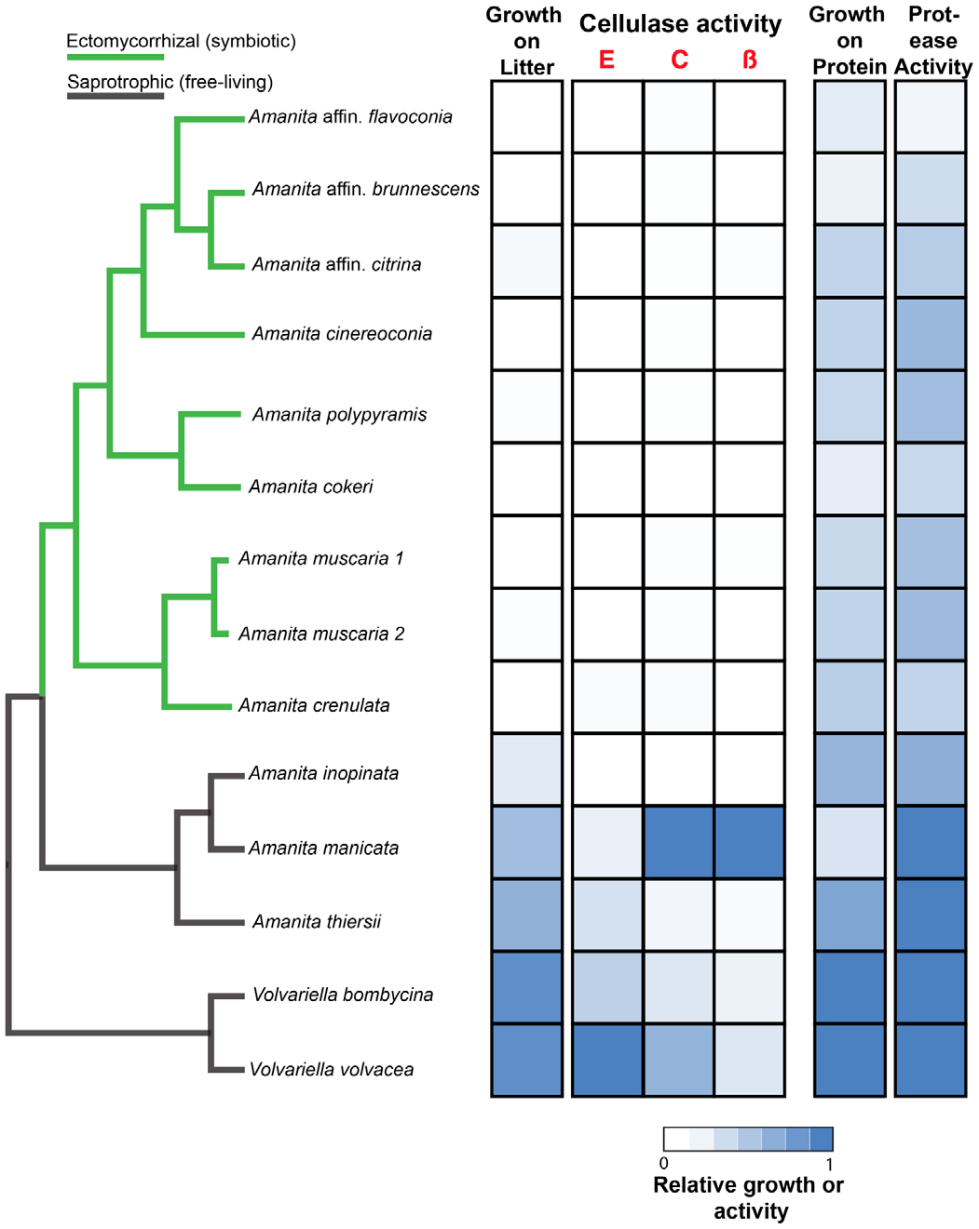
Experimental demonstration of the loss of cellulolytic saprotrophic capabilities by ectomycorrhizal *Amanita* species. Fourteen species, including saprotrophic and ectomycorrhizal *Amanita* and two saprotrophic *Volvariella* species, were grown on sterile grass litter to test for the use of litter as a sole carbon source. Enzyme activities of endoglucanase (E), cellobiohydrolase (C) and beta-glucosidase (ß) were also measured. The same species were grown on media with a protein (casein hydrolysate) as sole nitrogen source. Colony diameter as well as clearing of milk agar was measured to indicate growth on protein and protease activity, respectively. Values of cellulolytic and proteolytic growth and activity are expressed as relative to the maximum value (from 0 to 1) for each parameter across the species. The difference in growth between ectomycorrhizal and saprotrophic species on litter is highly significant, according to phylogenetically independent contrasts (F_1,11_ = 7.84, *p* = 0.017). Contrasts for all other data were not significant.

All three SAP *Amanita* species and the outgroup species grew on litter, while EM *Amanita* species did not grown on litter (Figure 4), confirming that obligately symbiotic species have lost the capacity for saprotrophic growth on complex cellulosic substrates. *Amanita inopinata*, a free-living species without the three cellulases, also grew very poorly on litter, confirming it is not an effective decomposer of cellulose. We also measured the production of endoglucanase, cellobiohydrolase, and beta-glucosidase by each species. While two SAP *Amanita* species (*A. manicata* and *A. thiersii*) and the SAP outgroups produced significant levels of all three enzymes, these enzymes were not detectable or were only produced in trace amounts by EM *Amanita* species (Figure 4). Despite having functional copies of *bgl*, beta-glucosidase activity was not detected from EM species, probably because these species grew so poorly on the litter.

In addition to carbon, EM fungi require high levels of nitrogen both for their own metabolism and as a resource for host plants. Many EM fungi, including some *Amanita* species, have been shown to use various organic nitrogen sources [45]. We predicted that while cellulolytic SAP capabilities would be lost by EM *Amanita*, proteolytic capabilities would be retained. Our hypothesis is supported by the ability of both SAP and EM *Amanita* to grow when protein was provided as the sole nitrogen source (Figure 4). Production of extracellular proteases was also detected from all *Amanita* species, confirming a capacity to degrade proteins in the soil environment (Figure 4 and S7). Partial sequences of an extracellular aspartic protease originally described from *Amanita muscaria* [46] were identified in both EM and SAP *Amanita* species (Figure S8) suggesting the genetic potential for proteolytic activity has been retained following the evolution of the EM symbiosis.

### Conclusions

Using the genus *Amanita*, we demonstrate that the irreversible loss of genes required for autonomous growth is associated with the evolution of an ectomycorrhizal symbiosis. We cannot determine if the loss of cellulases was a prerequisite for the evolution of the EM symbiosis in *Amanita*, or a consequence. However, the loss of genes required for autonomous growth is also observed with prokaryotic endosymbionts [1], [4], [12], suggesting that common patterns of genome evolution may occur with both endo- and ectosymbionts. Relaxed selection on genes enabling access to resources readily provided by hosts may be a common mechanism driving gene loss. Using phylogenetic, genomic, and experimental approaches with other microbial, eukaryotic ectosymbionts, such as fungal symbionts of leaf-cutting ants [47], [48] or bark beetles [49], may provide additional insights into patterns and mechanisms of gene loss associated with transitions to symbiosis.

The evolutionary lability of the EM symbiosis has been intensely debated since the first large-scale phylogeny of EM and SAP basidiomycete species suggested transitions from SAP to EM have occurred many times, and reversions from EM to SAP are also likely [50]. Subsequent work questioned the existence of reversals from EM to SAP niches [51], and pointed to the problems of using poorly resolved molecular phylogenies to draw conclusions about ecological transitions. As far as we are aware, our research is the first to combine fine-scale phylogenetic, functional gene, and experimental data to demonstrate that following the evolution of an EM symbiosis, reversions to SAP are unlikely. Ectomycorrhizal EM *Amanita* species and other EM fungi have not lost all genes associated with the decomposition of organic matter, as the retention of beta-glucosidases by EM *Amanita* demonstrates. However, because critical genes in decomposition pathways have been lost, it is unlikely that these fungi will be able to acquire significant amounts of carbon from saprotrophy and instead will rely on carbon provided by a host plant. Our data suggest that the limited ability of EM *Amanita* to easily decompose complex organic matter is a constraint on reversions to a SAP niche and is likely to play a key role in the maintenance of these widespread microbial mutualisms.

## Materials and Methods

### Phylogenetic and Ancestral Character Reconstruction

To reconstruct the evolutionary history of the EM symbiosis within *Amanita*, we used a four gene phylogeny of 108 species collected from around the world. Specimens were taken from herbaria or were collected by the authors from the wild (see table S1 for details on how specimens were obtained). All necessary permits for collecting were obtained and no endangered or protected species were collected. This is the most geographically and taxonomically diverse phylogeny of *Amanita* to date. Moreover, this is the first molecular phylogeny to include a significant sampling of the 32 probable SAP *Amanita* species, with 13 of these (Table S1) included in our work. Thirteen of the remaining SAP *Amanita* species were excluded because the known collections were judged too old or degraded to provide DNA. According to morphological characters [35], the 6 unsampled species are more closely related to other SAP *Amanita* than EM *Amanita*, and molecular phylogenies consistently support morphological groupings in *Amanita* [52], [53]. Several species of closely related SAP genera, including *Limacella*, *Volvariella*, and *Pluteus*, were also included.

We sequenced 4 gene regions, the nuclear and mitochondrial large and small subunits of rRNA, using standard primers (nucLSU: LR0R/LR7; nucSSU: PNS1/NS41; mtSSU: MS1/MS2; mtLSU: ML5/ML6). The final alignment after manually removing unalignable regions included 2994 characters for 108 species. To test for topological congruence among each of the 4 loci, separate maximum likelihood phylogenies were constructed for each gene using RaxML (version 7.2.3) with 100 bootstrap replicates. Using these single-locus phylogenies, we did not detect any well-supported (>80% bootstrap support) topological conflicts among genes, and therefore concatenated the genes into a single dataset. A GTR+I+Γ model of evolution was determined to be suitable for all 4 gene regions based on model selection analyses implemented in TOPALI version 2.5 [54].

Maximum likelihood analysis was performed with RA×ML version 7.2.3 [55], using a GTR+I+Γ model with 100 bootstrap replicates. Bayesian Analysis was performed with MrBayes version 3.1 using a GTR+I+Γ model with ten million generations, 4 chains, trees sampled every 1000 generations, and a burn-in period of 2.5 million generations. In both analyses, *Volvariella volvacea* was used as an outgroup. We repeated our phylogenetic analysis with other outgroup taxa that have been placed in the Pluteoid clade [13] including *Melanoleuca*, *Pluteus*, and *Pleurotus.* The choice of outgroup did not change the phylogenetic relationships we observed between EM and SAP *Amanita* taxa.

Maximum likelihood ancestral state reconstruction of trophic status (SAP vs. EM) was optimized onto the best maximum likelihood tree using parsimony and likelihood ancestral states in Mesquite version 2.73+ (mesquiteproject.org). For likelihood ancestral state reconstruction, a Markov k-state 1-parameter model (Mk1) was used with the likelihood decision threshold set to 2.

### Detection of Cellulase Genes

Using degenerate PCR primers designed for cellulase homologs of basidiomycetes (Table S2), we screened every species in our phylogeny for the presence of endoglucanase, cellobiohydrolase, and beta-glucosidase genes. Primers (Table S2) were designed based on *V. volvacea* sequences as well as other known sequences of genes from genomes of other basidiomycetes, with the exception of the primer for cellobiohydrolase I (*cbhI-I*), which was previously published [39]. When an amplicon of the expected size was detected, it was cloned using a TOPO TA Cloning Kit (Invitrogen, Carlsbad, CA). Cloned inserts were amplified from eight colonies per cloning reaction using colony PCR with M13 primers and sequenced.

Because the beta-glucosidase gene (*bgl*) was broadly distributed across the phylogeny, we only cloned and sequenced a subset of total detected amplicons. In initial screens, we could predict with 100% accuracy if a *bgl* amplicon was present in a species based on the presence of an abundant band ∼525 bp in length. When this band was not present, many faint bands with a diversity of sizes were present that were not homologous to *bgl* (based on cloning and sequencing), or no bands were present. To assist with direct sequencing of *bgl* when it was detected, we added M13 tags to both the forward and the reverse primers.

Sequences were aligned in MacClade. Deduced amino acid sequences were used to generate maximum likelihood phylogenies for each gene using RaxML with a JTT model of amino acid substitution and gamma rate heterogeneity. 100 bootstrap replicates were performed for determining node support.

To confirm that the lack of a PCR amplicon for a cellulase gene correctly predicted its absence from a genome, we performed Southern blots on a subset of species from the larger phylogeny. Five micrograms of genomic DNA (gDNA) extracted from axenically grown mycelium (see below) was digested with *Hind*III, run out on a 1% agarose gel, and transferred to a nylon membrane. Gel purified PCR products produced from amplification of *A. manicata* gDNA with the primers for each gene described above were used to generate probes for the Southern blots with ^32^P-dCTP using the Prime-It II kit (Stratagene, La Jolla, CA). Blots were hybridized overnight at 65°C and washed for 2×10 min with (4× SSPE/1% SDS) and 1×5 min in (2× SSPE/1% SDS) at 65°C. The membrane was exposed to a phosphor screen overnight and imaged on a Typhoon Imager. The same blot was used for each gene. The blot was washed with 0.1 M NaOH/0.2% SDS between each hydridization to remove the probe and neutralized with a 0.1× SSC/0.2% SDS/0.2 M Tris-Cl solution. A control gene, elongation factor 1-alpha, was used for a final hybridization reaction.

### Experimental Assessment of Saprotrophy

To experimentally assess SAP capabilities of EM species, we first grew strains of 14 taxa isolated from sporocarps (Table S3). Then a 2 mm×2 mm piece of mycelium from the actively growing front of a culture growing on potato dextrose agar was added to the center of a 60 mm Petri dish containing one of two treatments: solid medium with just basal salts and no carbon source (to control for residual growth from stored carbohydrates) and solid medium with basal salts as well as sterile grass litter. Each species and treatment combination was replicated 5 times. The grass litter comprised four species: *Panicum virgatum*, *Elymus canadensis*, *Andropogon gerardii*, and *Koeleria cristata*. We used grass litter because it was easy to grow in a controlled sterile environment and because many free-living *Amanita* species grow in grassland habitats. However, similar results were observed in a pilot study using sterile wood chips and artificial cellulose (Sigmacell and carboxymethylcellulose). Grasses were grown from seed in potting soil in a controlled environment growth chamber (20/25 degrees C, 12 h day/night periods, 60% humidity, and 500 µmol photon m^−2^ s^−1^ illumination). After 1 month of growth, we stopped watering the plants to cause senescence. Plants were harvested and cut into 2–3 cm segments and mixed in equal proportions for each of the four species. Grass litter was steam sterilized twice, at 121°C for 20 minutes, with a rinse in sterile water between autoclaving sessions to help remove pigments that might interfere with enzyme assays (see below).

We then assayed the activity of extracellular endoglucanase, cellobiohydrolase, and beta-glucosidase of fungi growing in liquid medium with sterile grass litter. Fungi were cultured in exactly the same manner used for the saprotrophy assay, except that liquid medium was used and only the litter treatment was used. To assay enzyme activity, 50 µL of liquid was removed from directly under the actively growing hyphal front of a mycelium. Each species was replicated 4 times. Endoglucanase activity was measured by determining the amount of reduced sugars produced after incubation of the culture liquid with carboxymethylcellulose. Reduced sugar concentrations were determined using the Somogyi-Nelson method [56], with glucose as a standard. Cellobiohydrolase was assayed by measuring the release of *p*-nitrophenol from *p*-nitrophenyl-β-d-cellobioside and beta-glucosidase was assayed by measuring the release of *p*-nitrophenol from *p*-nitrophenol–glucopyranoside. Substrate controls were included to account for differences in the release of pigments, nutrients or other compounds from the litter that could interfere with spectrophotometric assays. Water blanks were also used to account for background. Enzyme activity was measured at three time points (4, 16 and 32 days), as peak enzyme activity of the fungi varied in pilot experiments. Data presented is the mean value across the three time points. Endoglucanase is expressed as amount of reduced sugars produced. Cellobiohydrolase and beta-glucosidase is expressed as the amount of p-nitrophenol released from the artificial substrates.

Growth on protein and protease activity was measured as previously described [57]. Hyphal diameter was used to quantify growth on protein and the size of the halo of clearing on milk agar plates was used to quantify protease activity. Aspartic protease genes were amplified using degenerate primers designed using sequences of aspartic protease retrieved from basidiomycete genomes (Table S2).

To test for significant differences in growth on cellulose or protein as well as cellulase and protease activity, we used phylogenetically independent contrasts [58]. We tested for differences between SAP species (*Volvariella* and the three *Amanita* species) and the EM *Amanita* species for each of the cellulotyic or proteolytic variables measured. Independent contrasts were conducted using PDAP version 1.15 implemented in Mesquite version 2.73. A phylogeny of the nucLSU data was used for branch lengths.

### Beta-glucosidase Expression

To determine levels of expression of *bgl* by *Amanita* species, cultures of three species, *A. thiersii*, *A. cokeri* and *A. crenulata*, were grown with media using the same basal salts as described above with either 2% cellobiose or 2% glucose as sole carbon sources. Both cellobiose and glucose were filter sterilized to prevent degradation of cellobiose through heat sterilization. To determine transcript abundance of *bgl*, RNA was extracted using an RNeasy Plant Mini Kit (Qiagen Valencia, California). cDNA was constructed from the RNA using a Qiagen Quantitect Reverse Transcription Kit and used for PCR using gene specific primers (Table S2).

## Supporting Information

Figure S1 **Maximum likelihood phylogeny of endoglucanase orthologs.** Sequences were obtained by PCR amplification from saprotrophic *Amanita* species and close saprotrophic relatives to *Amanita* (*Volvariella*, *Limacella* and *Pluteus*) or were retrieved from genomes of other saprotrophic fungi from NCBI. The phylogeny is based on an amino-acid alignment of 156 characters. Values indicate bootstrap support. Support values are indicated for nodes with ≥50 bootstrap support.
(DOC)

Figure S2 **Maximum likelihood phylogeny of cellobiohydrolase I orthologs.** Sequences were obtained by PCR amplification from saprotrophic *Amanita* species and close saprotrophic relatives to *Amanita* (*Volvariella*, *Limacella* and *Pluteus*) or were retrieved from genomes of other saprotrophic fungi from NCBI. The phylogeny is based on an amino-acid alignment of 172 characters. Values indicate bootstrap support. Support values are indicated for nodes with ≥50 bootstrap support.
(DOC)

Figure S3 **Southern blots of various** ***Amanita*** **species.** (A) Genomic DNA digested with *Hind*III. (B) Endoglucanase, (C) Cellobiohydrolase I, and (D) a control probe of elongation factor 1-alpha. The same membrane was reprobed in (B) through (D). Species are: Lane 1) *Amanita manicata*, 2) *A. thiersii*, 3) *A. inopinata*, 4) *A. cokeri* 5) *A. cinereoconia*, 6) *A. citrina* 7) *A.* affin *flavoconia* 8) *A. crenulata* 9) *A. muscaria* var. *guessowii*. In each blot, hybridizations were done using PCR product generated from *A. manicata* (lane 1).
(DOC)

Figure S4 **Maximum likelihood phylogeny of beta-glucosidase** **orthologs.** Sequences were obtained by PCR amplification from saprotrophic and ectomycorrhizal *Amanita* species and close saprotrophic relatives to *Amanita* (*Volvariella*, *Limacella* and *Pluteus*) or were retrieved from genomes of other saprotrophic fungi from NCBI. Phylogeny is based on an amino-acid alignment of 117 characters. Values indicate bootstrap support. Support values are indicated for nodes with ≥50 bootstrap support.
(DOC)

Figure S5 **Root morphology of** ***Picea abies*** **seedlings following inoculation with various** ***Amanita*** **species.** **(A)** No inoculation, **(B)** *A. thiersii* (saprotrophic), **(C)** *A. inopinata* (saprotrophic) and **(D)** *A. muscaria* (ectomycorrhizal).
(DOC)

Figure S6 **PCR amplicons of putative cellobiohydrolases from species spanning sister saprotrophic and ectomycorrhizal clades.** Invitrogen Low Mass Ladder in first and last lane. Amplicons are shown in lanes 1–23 and were amplified from gDNA of the following specimens from the Farlow Herbarium (FH) at Harvard: 1) *Hebeloma* sp. (FH 00300668), 2) *Hebeloma circinans* (FH 00286370), 3) *Hebeloma pusilum* (FH 00300671), 4) *Hebeloma radicatum* (FH 00300672), 5) *Hebeloma crustuliniforme* (FH D-352), 6) *Galerina* sp. (FH 00300674), 7) *Galerina calyptrate* (FH 00300675), 8) *Galerina marginata* (FH 00286349), 9) *Galerina paludosa* (FH D-159), 10) *Galerina tibiicystis* (FH D-160), 11) *Psilocybe* cf. *coprophila* (FH 00284978), 12) *Tricholoma quericola* (FH 00284932), 13) *Tricholoma vaccinum* (FH 00304011), 14) *Tricholoma atrosquamosum* (FH 00286508), 15) *Tricholoma orirubens* (FH Yang 2323), 16) *Tricholoma saponaceum* (FH 00286488), 17) *Tricholoma squarrulosum* (FH Ge 347), 18) *Tricholoma sulphureum* (FH Ge 864), 19) *Lepista nuda* (FH 00300634), 20) *Lepista inversa* (FH 00300633), 21) *Lepista flaccida* (FH 00286364), 22) *Clitocybe gibba* (FH Ge 588). Amplicons in lanes 3, 13 and 17 were cloned and sequenced and showed high similarity to basidiomycete cellobiohydrolases. Other bands amplified from ectomycorrhizal species were found to be due to non-specific amplification.
(DOC)

Figure S7 **Photos of representative plates from the litter (first column of photos) and protein (second column of photos) growth experiments.** Halos around cultures on protein plates indicate protease activity. Only *Amanita* species are shown.
(DOC)

Figure S8 **Maximum likelihood phylogeny of aspartic protease orthologs.** Sequences were obtained by PCR amplification from saprotrophic and ectomycorrhizal *Amanita* species and close saprotrophic relatives to *Amanita* (*Volvariella*, *Limacella* and *Pluteus*) or were retrieved from genomes of other saprotrophic fungi from NCBI. Phylogeny is based on an amino-acid alignment of 182 characters. Values indicate bootstrap support. Support values are indicated for nodes with ≥50 bootstrap support.
(DOC)

Table S1 Specimens of *Amanita* and close relatives used to construct phylogeny.
(PDF)

Table S2 **Primers used to amplify functional genes.**
(PDF)

Table S3 **Cultures used in experimental assessment of saprotrophy.**
(PDF)
