# Supplementary material for: The Irreversible Loss of a Decomposition Pathway Marks the Single Origin of an Ectomycorrhizal Symbiosis
Source: PLoS One. 2012 Jul 18;7(7):e39597. doi: 10.1371/journal.pone.0039597 (PMC3399872; doi:10.1371/journal.pone.0039597)
Supplement: Figure S2 — Maximum likelihood phylogeny of cellobiohydrolase I orthologs. Sequences were obtained by PCR amplification from saprotrophic Amanita species and close saprotrophic relatives to Amanita (Volvariella, Limacella and Pluteus) or were retrieved from genomes of other saprotrophic fungi from NCBI. The phylogeny is based on an amino-acid alignment of 172 characters. Values indicate bootstrap support. Support values are indicated for nodes with ≥50 bootstrap support. (DOC) [file pone.0039597.s002.doc]

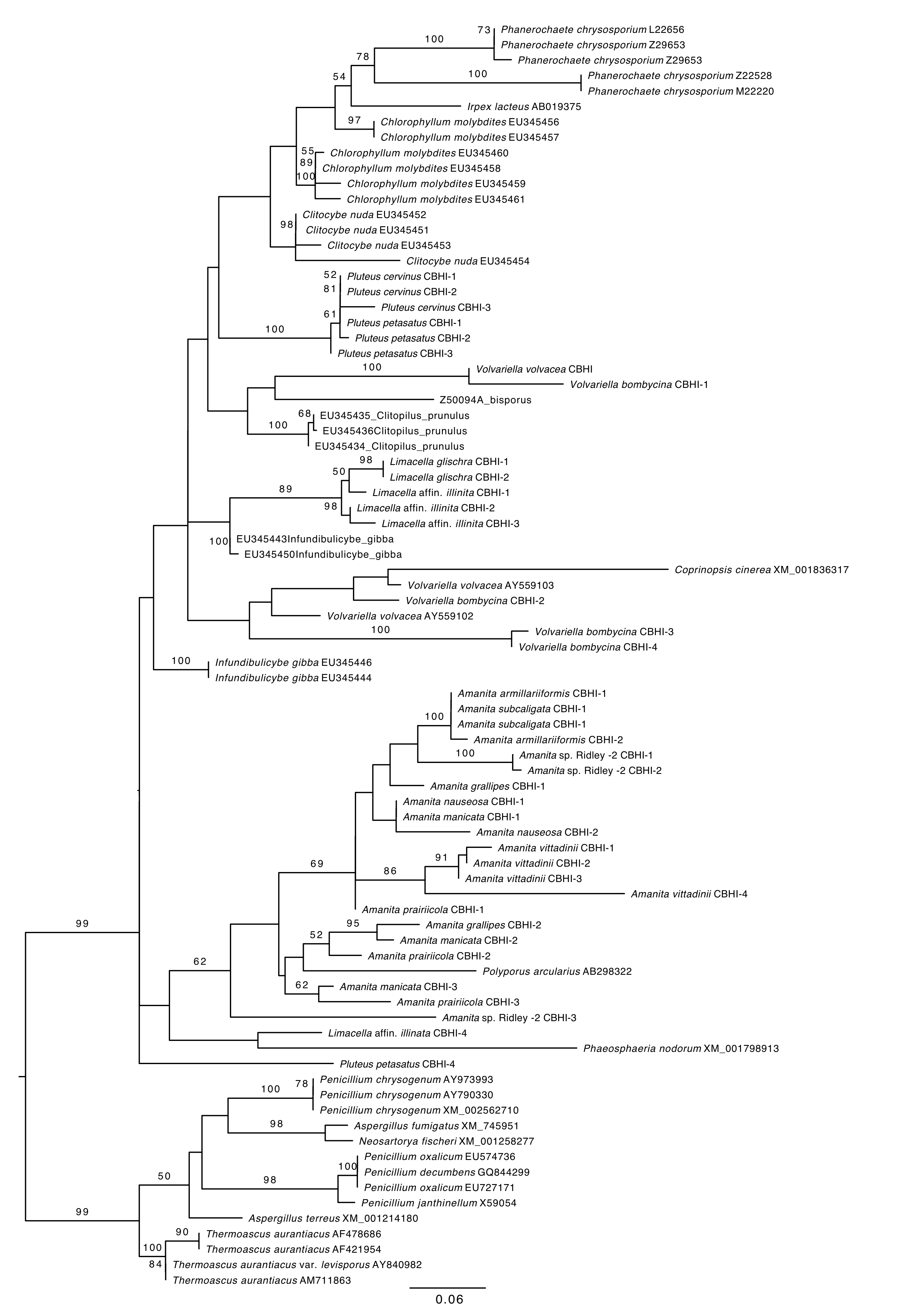


**Figure S2:** **Maximum likelihood phylogeny of cellobiohydrolase I orthologs.** Sequences were obtained by PCR amplification from saprotrophic *Amanita* species and close saprotrophic relatives to *Amanita* (*Volvariella*, *Limacella* and *Pluteus*) or were retrieved from genomes of other saprotrophic fungi from NCBI. The phylogeny is based on an amino-acid alignment of 172 characters. Values indicate bootstrap support. Support values are indicated for nodes with ≥ 50 bootstrap support.
