# Supplementary material for: The Irreversible Loss of a Decomposition Pathway Marks the Single Origin of an Ectomycorrhizal Symbiosis
Source: PLoS One. 2012 Jul 18;7(7):e39597. doi: 10.1371/journal.pone.0039597 (PMC3399872; doi:10.1371/journal.pone.0039597)
Supplement: Figure S3 — Southern blots of various Amanita species. (A) Genomic DNA digested with HindIII. (B) Endoglucanase, (C) Cellobiohydrolase I, and (D) a control probe of elongation factor 1-alpha. The same membrane was reprobed in (B) through (D). Species are: Lane 1) Amanita manicata, 2) A. thiersii, 3) A. inopinata, 4) A. cokeri 5) A. cinereoconia, 6) A. citrina 7) A. affin flavoconia 8) A. crenulata 9) A. muscaria var. guessowii. In each blot, hybridizations were done using PCR product generated from A. manicata (lane 1). (DOC) [file pone.0039597.s003.doc]

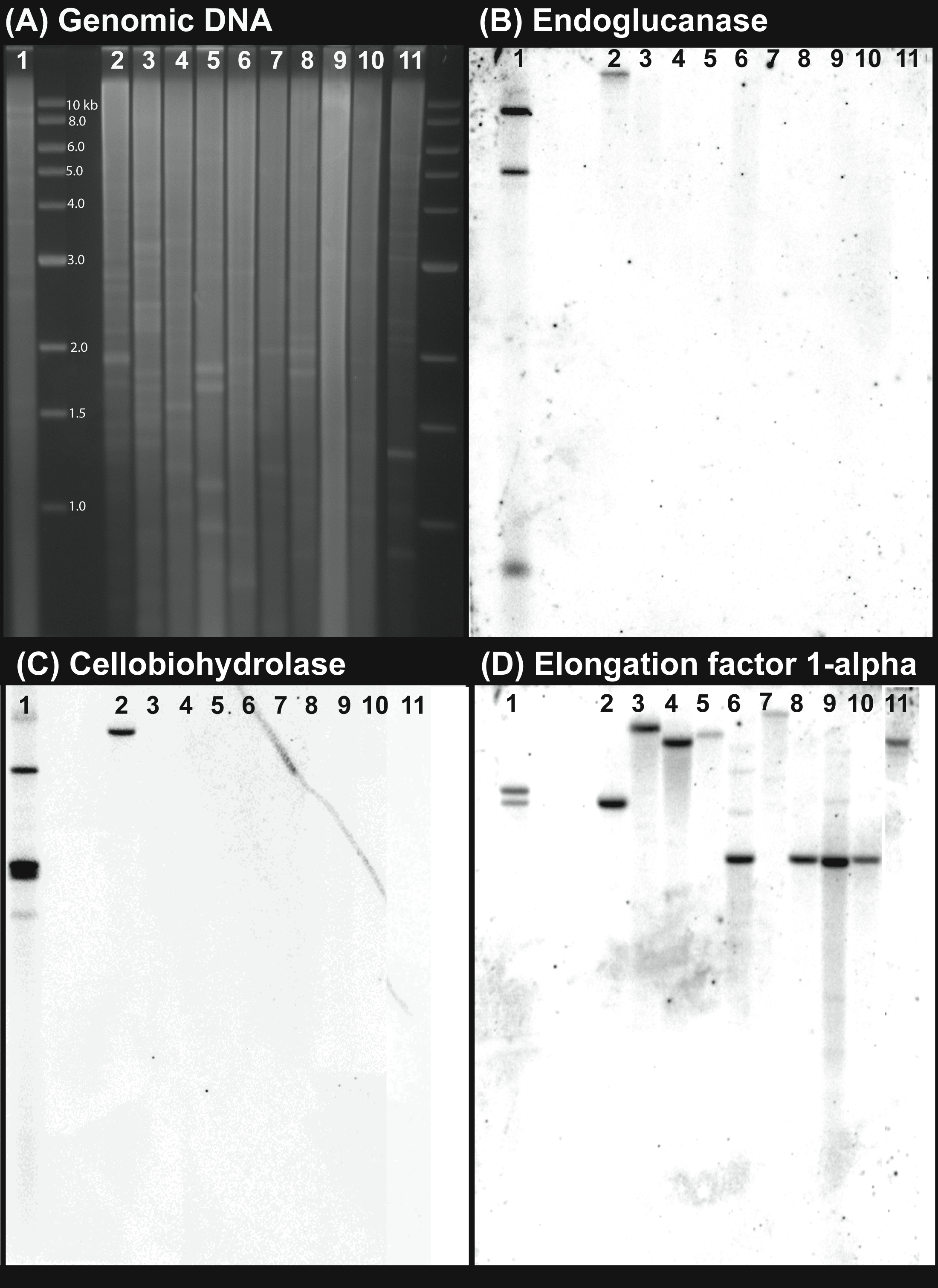


**Figure S3:** **Southern blots of various *Amanita* species.** (A) Genomic DNA digested with *Hind*III. (B) Endoglucanase, (C) Cellobiohydrolase I, and (D) a control probe of elongation factor 1-alpha. The same membrane was reprobed in (B) through (D). Species are: Lane 1) *Amanita manicata*, 2) *A. thiersii*, 3) *A. inopinata*, 4) *A. cokeri* 5) *A. cinereoconia*, 6) *A. citrina* 7) *A.* affin *flavoconia* 8) *A. crenulata* 9) *A. muscaria* var. *guessowii*. In each blot, hybridizations were done using PCR product generated from *A. manicata* (lane 1).
