# Supplementary material for: The Irreversible Loss of a Decomposition Pathway Marks the Single Origin of an Ectomycorrhizal Symbiosis
Source: PLoS One. 2012 Jul 18;7(7):e39597. doi: 10.1371/journal.pone.0039597 (PMC3399872; doi:10.1371/journal.pone.0039597)
Supplement: Figure S5 — Root morphology of Picea abies seedlings following inoculation with various Amanita species. (A) No inoculation, (B) A. thiersii (saprotrophic), (C) A. inopinata (saprotrophic) and (D) A. muscaria (ectomycorrhizal). (DOC) [file pone.0039597.s005.doc]

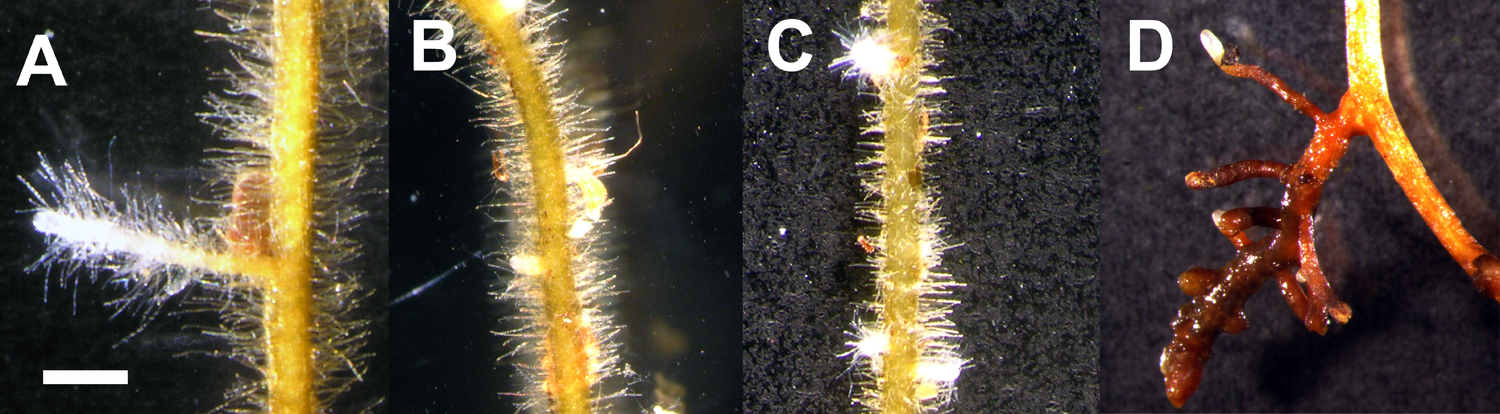


**Figure S5:** **Root morphology of *Picea abies* seedlings following inoculation with various *Amanita* species.** **(A)** No inoculation, **(B)** *A. thiersii* (saprotrophic), **(C)** *A. inopinata* (saprotrophic) and **(D)** *A. muscaria* (ectomycorrhizal).
