# Supplementary material for: The Irreversible Loss of a Decomposition Pathway Marks the Single Origin of an Ectomycorrhizal Symbiosis
Source: PLoS One. 2012 Jul 18;7(7):e39597. doi: 10.1371/journal.pone.0039597 (PMC3399872; doi:10.1371/journal.pone.0039597)
Supplement: Figure S6 — PCR amplicons of putative cellobiohydrolases from species spanning sister saprotrophic and ectomycorrhizal clades. Invitrogen Low Mass Ladder in first and last lane. Amplicons are shown in lanes 1–23 and were amplified from gDNA of the following specimens from the Farlow Herbarium (FH) at Harvard: 1) Hebeloma sp. (FH 00300668), 2) Hebeloma circinans (FH 00286370), 3) Hebeloma pusilum (FH 00300671), 4) Hebeloma radicatum (FH 00300672), 5) Hebeloma crustuliniforme (FH D-352), 6) Galerina sp. (FH 00300674), 7) Galerina calyptrate (FH 00300675), 8) Galerina marginata (FH 00286349), 9) Galerina paludosa (FH D-159), 10) Galerina tibiicystis (FH D-160), 11) Psilocybe cf. coprophila (FH 00284978), 12) Tricholoma quericola (FH 00284932), 13) Tricholoma vaccinum (FH 00304011), 14) Tricholoma atrosquamosum (FH 00286508), 15) Tricholoma orirubens (FH Yang 2323), 16) Tricholoma saponaceum (FH 00286488), 17) Tricholoma squarrulosum (FH Ge 347), 18) Tricholoma sulphureum (FH Ge 864), 19) Lepista nuda (FH 00300634), 20) Lepista inversa (FH 00300633), 21) Lepista flaccida (FH 00286364), 22) Clitocybe gibba (FH Ge 588). Amplicons in lanes 3, 13 and 17 were cloned and sequenced and showed high similarity to basidiomycete cellobiohydrolases. Other bands amplified from ectomycorrhizal species were found to be due to non-specific amplification. (DOC) [file pone.0039597.s006.doc]

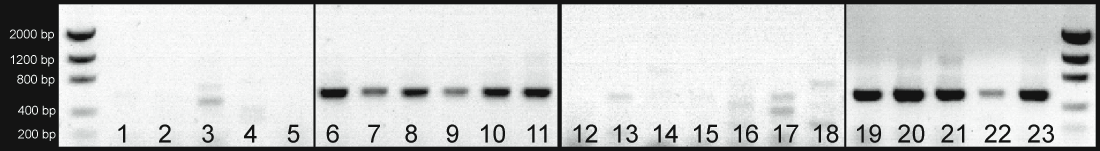


**Figure S6: PCR amplicons of putative cellobiohydrolases from species spanning sister saprotrophic and ectomycorrhizal clades.** Invitrogen Low Mass Ladder in first and last lane. Amplicons are shown in lanes 1-23 and were amplified from gDNA of the following specimens from the Farlow Herbarium (FH) at Harvard: 1) *Hebeloma* sp. (FH 00300668), 2) *Hebeloma* *circinans* (FH 00286370)*,* 3) *Hebeloma pusilum* (FH 00300671)*,* 4) *Hebeloma radicatum* (FH 00300672)*,* 5) *Hebeloma crustuliniforme* (FH D-352)*,* 6) *Galerina* sp. (FH 00300674), 7) *Galerina calyptrate* (FH 00300675)*,* 8) *Galerina marginata* (FH 00286349)*,* 9) *Galerina paludosa* (FH D-159)*,* 10) *Galerina tibiicystis* (FH D-160), 11) *Psilocybe* cf. *coprophila* (FH 00284978)*,* 12) *Tricholoma quericola* (FH 00284932)*,* 13) *Tricholoma vaccinum* (FH 00304011)*,* 14) *Tricholoma atrosquamosum* (FH 00286508)*,* 15) *Tricholoma orirubens* (FH Yang 2323)*,* 16) *Tricholoma saponaceum* (FH 00286488)*,* 17) *Tricholoma squarrulosum* (FH Ge 347)*,* 18) *Tricholoma sulphureum* (FH Ge 864)*,* 19) *Lepista nuda* (FH 00300634), 20) *Lepista inversa* (FH 00300633), 21) *Lepista flaccida* (FH 00286364), 22) *Clitocybe gibba* (FH Ge 588). Amplicons in lanes 3, 13 and 17 were cloned and sequenced and showed high similarity to basidiomycete cellobiohydrolases. Other bands amplified from ectomycorrhizal species were found to be due to non-specific amplification.
