# Supplementary material for: The Irreversible Loss of a Decomposition Pathway Marks the Single Origin of an Ectomycorrhizal Symbiosis
Source: PLoS One. 2012 Jul 18;7(7):e39597. doi: 10.1371/journal.pone.0039597 (PMC3399872; doi:10.1371/journal.pone.0039597)
Supplement: Figure S7 — Photos of representative plates from the litter (first column of photos) and protein (second column of photos) growth experiments. Halos around cultures on protein plates indicate protease activity. Only Amanita species are shown. (DOC) [file pone.0039597.s007.doc]

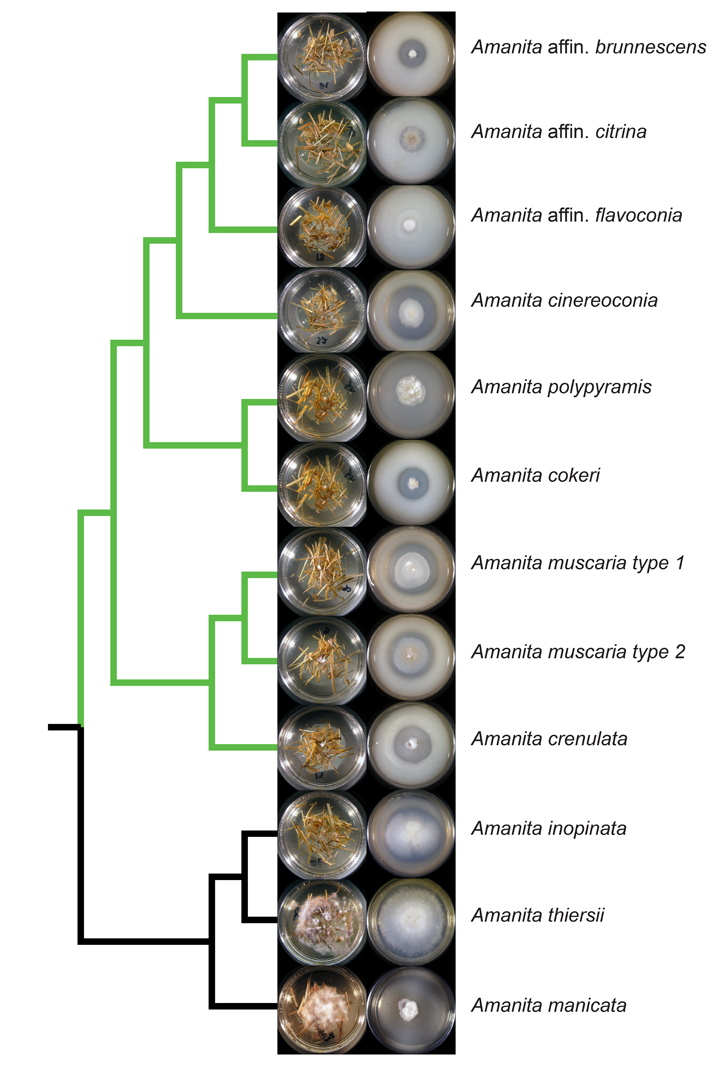


**Figure S7:** **Photos of representative plates from the litter (first column of photos) and protein (second column of photos) growth experiments.** Halos around cultures on protein plates indicate protease activity. Only *Amanita* (not *Volvariella*) species are shown.
