# Supplementary material for: The Irreversible Loss of a Decomposition Pathway Marks the Single Origin of an Ectomycorrhizal Symbiosis
Source: PLoS One. 2012 Jul 18;7(7):e39597. doi: 10.1371/journal.pone.0039597 (PMC3399872; doi:10.1371/journal.pone.0039597)
Supplement: Table S1 — Specimens of Amanita and close relatives used to construct phylogeny. (PDF) [file pone.0039597.s009.pdf]

Table S1: Specimens of *Amanita* and close relatives used in to construct phylogeny

Species	Symbiotic (SYM)/ Free-living (FL**)	Herbarium ID	Collection Location*	GenBank Accession #s			
				nucLSU	nucSSU	mtLSU	mtSSU
Amanita abrupta	SYM	BW_HP_101***	United States, Massachusetts	HQ539660	HQ539768	HQ539978	HQ539871
A. afrospinosa	SYM	RET 347-1	Zimbabwe, Harare	HQ539666	HQ539774	HQ539981	HQ539877
A. altipes	SYM	Yang 2431	China, Sichuan	HQ539667	HQ539775	HQ539982	HQ539878
A. amanitoides	SYM	RET 344-9	Zambia, Copper Belt Province	HQ539668	HQ539983	HQ539879	
A. armillariiformis	FL	RET 266-8	United States, Colorado	HQ539669	HQ539776	HQ539984	HQ539880
A. sp-Arora-01-560	SYM	RET 345-5	Zambia, Copper Belt Province	HQ539698	HQ539802	HQ540012	HQ539909
A. atkinsoniana	SYM	RET 301-1	United States, Connecticut	HQ539670	HQ539777	HQ539985	HQ539881
A. banningiana	SYM	BW_Shopkins#16	United States, New Jersey	HQ539671	HQ539778	HQ539986	HQ539882
A. brunnescens	SYM	BW_HP12**	United States, Massachusetts	HQ539674	HQ539780	HQ539989	HQ539885
A. affin. *brunnescens*	SYM	BW_HF 10C***	United States, Massachusetts	HQ539661	HQ539769	HQ539872	
A. brunneolocularis	SYM	NY 460913	Costa Rica, San José	HQ539673	HQ539779	HQ539988	HQ539884
A. cecilieae sensu auct. amer.	SYM	BW_HF071106-1***	United States, MA	HQ539675		HQ539990	HQ539886
A. chlorinosma	SYM	RET 328-6	United States, New York	HQ539676	HQ539781	HQ539991	HQ539887
A. cinereoconia	SYM	BW_PSF	United States, New Hampshire	HQ539677			HQ539888
A. cinereopannosa	SYM	RET 319-8	United States, Maine	HQ539678	HQ539782	HQ539992	HQ539889

A. citrina sensu auct. amer.	SYM	BW JLR 102106-1**	United States, New Jersey	HQ539679	HQ539783	HQ539993	HQ539890
A. affin. *citrina*	SYM	BW_PNC	United States, New Jersey	HQ539662	HQ539770		HQ539873
A. clelandii	SYM	PSC 2524	Australia, South Australia	HQ539680	HQ539784	HQ539994	HQ539891
A. codinae	FL	RET 275-8	France, Maine-et-Loire	HQ539681	HQ539785	HQ539995	HQ539892
A. cokeri	SYM	BW_STF 090506-19***	United States, Massachusetts	HQ539682	HQ539786	HQ539996	HQ539893
A. conicobulbosa	SYM	PSC 1368	Australia, South Australia	HQ539683	HQ539787	HQ539997	HQ539894
A. constricta	SYM	BW_Mycoblitz IV.2	United States, California	HQ539684	HQ539788	HQ539998	HQ539895
A. costaricensis	SYM	NY Halling 7684	Costa Rica, Cartago Province	HQ539685	HQ539789	HQ539999	HQ539896
A. crassiconus Bas nom. prov.	SYM	RET 348-5	Zambia, Copper Belt Province	HQ539686	HQ539790	HQ540000	HQ539897
A. crenulata	SYM	BW_PH82906-1***	United States, Massachusetts	HQ539687	HQ539791	HQ540001	HQ539898
A. daucipes	SYM	RET 386-8	United States, Pennsylvania	HQ539688	HQ539792	HQ540002	HQ539899
A. effusa	SYM	PSC 2007	Australia, South Australia	HQ539689	HQ539793	HQ540003	HQ539900
A. egregia	SYM	NY 66253	Australia, Queensland	HQ539690	HQ539794	HQ540004	HQ539901
A. eriophora	SYM	RET 350-4	Cambodia, Angkor	HQ539672	HQ539987	HQ539883	
A. affin. *excelsa*	SYM	Ge 816	China, Sichuan	HQ539691	HQ539795	HQ540005	HQ539902
A. farinacea	SYM	PSC 2529	Australia, South Australia	HQ539692	HQ539796	HQ540006	HQ539903
A. flavoconia	SYM	BW_PH22***	United States, Massachusetts	HQ539693	HQ539797	HQ540007	HQ539904
A. affin. *flavoconia*	SYM	PSMCC, #121	United States, Oregon	HQ539663	HQ539771		HQ539874
A. affin. flavorubens	SYM	BW_HF-FR***	United States, Massachusetts	HQ539664	HQ539772	HQ539979	HQ539875

A. flavorubens	SYM	RET 295-9	United States, New Jersey	HQ539694	HQ539798	HQ540008	HQ539905
A. friabilis	SYM	PAM 06082601	France, Savoie	HQ539695	HQ539799	HQ540009	HQ539906
A. frostiana	SYM	BW_PH37**	United States, Massachusetts	HQ539696	HQ539800	HQ540010	HQ539907
A. fulva sensu auct. amer..	SYM	BW_PH82906-7**	United States, MA	HQ539697	HQ539801	HQ540011	HQ539908
A. gracilior	SYM	RET 342-2	France, Charente-Maritime	HQ539699	HQ539803	HQ540013	HQ539910
A. grallipes	FL	RET 379-5	Brazil, Paraná State	HQ539700	HQ539804	HQ540014	HQ539911
A. hesleri	SYM	RET 155-1	United States, Mississippi	HQ539701	HQ539805	HQ540015	HQ539912
A. inopinata	FL	BW_GK2005-1	United Kingdom, Suffolk	HQ539702	HQ539806	HQ540016	HQ539913
A. jacksonii	SYM	BW_PH17**	United States, MA	HQ539703	HQ539807	HQ540017	HQ539914
A. longipes	SYM	RET 360-1	United States, New Jersey	HQ539704	HQ539808	HQ540018	HQ539915
A. affin. *luteofusca*	SYM	PSC 1093b	Australia, South Australia	HQ539705	HQ539809	HQ539916	
A. affin. *luteolovelata*	SYM	PSC 2187	Australia, South Australia	HQ539706	HQ539810	HQ539917	
A. manicata	FL	RET 387-4	United States, Hawaii	HQ539708	HQ539812	HQ540020	HQ539919
A. magniverrucata	SYM	RET 387-10	United States, California	HQ539707	HQ539811	HQ540019	HQ539918
A. marmorata	SYM	AP_RoweAM2005	United States, Hawaii	HQ539709	HQ539813	HQ540021	HQ539920
A. multisquamosa	SYM	BW_RP14**	United States, New York	HQ539710	HQ539814	HQ540022	HQ539921
A. murinoflammeum	SYM	NY 66697	Australia, Australian Capital Territory	HQ539711	HQ539815	HQ540023	HQ539922
A. muscaria subsp. *flavivolvata*	SYM	BW_Viess_01	United States, California	HQ539712	HQ539816	HQ540024	HQ539923
A. muscaria var. guessowii	SYM	BW_STF090506-7	United States, Massachusetts	HQ539713	HQ539817	HQ539924	

A. mutabilis	SYM	RET 314-6	United States, Florida	HQ539714	HQ539818	HQ540025	HQ539925
A. nauseosa	FL	DPL 6117	United States, Texas	HQ539715	HQ539819	HQ540026	HQ539926
A. novinupta	SYM	RET 093-10	United States, Oregon	HQ539716	HQ539820	HQ540027	HQ539927
A. ochrophylla	SYM	PSC 1127	Australia, South Australia, S. Australia	HQ539717	HQ539821	HQ540028	HQ539928
A. onusta	SYM	RET 297-3	United States, New Jersey	HQ539718	HQ539822	HQ540029	HQ539929
A. parvipantherina	SYM	Yang 2490	China, Yunnan	HQ539719	HQ539823	HQ540030	HQ539930
A. peckiana	SYM	RET 320-3	United States, New York	HQ539720	HQ539824	HQ540031	HQ539931
A. pelioma	SYM	RET 321-4	United States, Texas	HQ539721	HQ539825	HQ540032	HQ539932
A. phalloides	SYM	BW_JLR0226***	United States, New Jersey	HQ539722	HQ539826	HQ540033	HQ539933
A. polypyramis	SYM	RET 159-8	United States, Maryland	HQ539723	HQ539827	HQ540034	HQ539934
A. populiphila	SYM	RET 266-9	United States, Colorado	HQ539724	HQ539828	HQ540035	HQ539935
A. praecox Lamoureux nom. prov.	SYM	BW_PH082906-9***	United States, Massachusetts	HQ539725	HQ539829	HQ540036	HQ539936
A. praelongispora	SYM	RET 314-8	United States, Florida	HQ539726	HQ539830	HQ540037	HQ539937
A. prairiicola	FL	RET 266-1	United States, Colorado	HQ539727	HQ539831	HQ540038	HQ539938
A. proxima	SYM	RET 290-10	France, Gironde	HQ539728	HQ539832	HQ540039	HQ539939
A. pruitii	FL	RET 343-2	United States, Oregon	HQ539729	HQ539833	HQ540040	HQ539940
A. pudica	SYM	RET 344-4	Zambia, Copper Belt Province	HQ539730	HQ539834	HQ540041	HQ539941
A. pulverulenta	SYM	RET 348-9	Zambia, Northern Province	HQ539731	HQ539835	HQ540042	HQ539942

A. pyramidiferina	SYM	PSC 2441	Australia, South Australia	HQ539732	HQ539836	HQ540043	HQ539943
A. rhopalopus	SYM	RET 386-3	United States, West Virginia	HQ539733	HQ539837	HQ540044	HQ539944
A. sp-Ridley-2	FL	PDD 93780	New Zealand, Hawke's Bay	HQ539742	HQ539846	HQ540053	HQ539953
A. ristichii	SYM	BW_FR2_2006	United States, Massachusetts	HQ539734	HQ539838	HQ540045	HQ539945
A. rubescens	SYM	RET 122-8	Turkey, Trabzon	HQ539735	HQ539839	HQ540046	HQ539946
A. rubescens var. congolensis	SYM	RET 346-6	Zambia, Copper Belt Province	HQ539736	HQ539840	HQ540047	HQ539947
A. silvicola	SYM	RET 382-4	United States, California	HQ539737	HQ539841	HQ540048	HQ539948
A. singeri	FL	LIP 88.056	France, Vendée	HQ539738	HQ539842	HQ540049	HQ539949
A. sinicoflava	SYM	BW_PH061306-1***	United States, Massachusetts	HQ539739	HQ539843	HQ540050	HQ539950
A. smithiana	SYM	RET 382-6	United States, California	HQ539740	HQ539844	HQ540051	HQ539951
A. solitaria	SYM	RET 298-1	France, Gironde	HQ539741	HQ539845	HQ540052	HQ539952
A. affin. *spissa*	SYM	NY 47779	Germany, Bavaria	HQ539743	HQ539847	HQ540054	HQ539954
A. strobiliformis	SYM	AP_10407-1	Serbia, Belgrade	HQ539744	HQ539848	HQ540055	HQ539955
A. subalbida	SYM	PSC 2509	Australia, South Australia	HQ539745	HQ539849	HQ540056	HQ539956
A. subcaligata	FL	RET 266-6	United States, Colorado	HQ539746	HQ539850	HQ540057	HQ539957
A. subcokeri Tulloss nom. prov.	SYM	RET 097-3	United States, New Jersey	HQ539747	HQ539851	HQ540058	HQ539958
A. subglobosa	SYM	Yang 2488	China, Yunnan	HQ539748	HQ539852	HQ540059	HQ539959
A. sublutea	SYM	PSC 2401	Australia, South Australia	HQ539749	HQ539853	HQ540060	HQ539960
A. subsolitaria	SYM	RET 327-4	United States, New Jersey	HQ539750	HQ539854	HQ540061	HQ539961

A. tephrea Bas nom. prov.	SYM	RET 378-9	United States, New York	HQ539751	HQ539855	HQ540062	HQ539962
A. thiersii	FL	DPL 7272	United States, Missouri	HQ539752	HQ539856	HQ540063	HQ539963
A. umbrinella	SYM	PSC 1813	Australia, South Australia	HQ539753	HQ539857	HQ540064	HQ539964
A. affin. *vaginata*	SYM	BW_PH82906-11***	United States, Massachusetts	HQ539754	HQ539858	HQ540065	HQ539965
A. verna	SYM	RET 280-3	France, Gironde	HQ539755	HQ539859	HQ540066	
A. affin. *virosa*	SYM	BW_HP082906-2***	United States, Massachusetts	HQ539756	HQ539860	HQ540067	HQ539966
A. vittadinii	FL	RET 277-1	France, Maine-et-Loire	HQ539757	HQ540068	HQ539967	
A. affin. *volvata*	SYM	BW_STF 090506-8***	United States, Massachusetts	HQ539665	HQ539773	HQ539980	HQ539876
A. wellsii	SYM	RET 001-5	United States, Maine	HQ539758	HQ539861	HQ540069	HQ539968
A. westii	SYM	BW_SH26	United States, Texas	HQ539759	HQ539862	HQ540070	HQ539969
A. xylinivolva	SYM	NY 460959	Costa Rica, San José	HQ539760	HQ539863	HQ540071	HQ539970
A. yuaniana	SYM	Yang 2489	China, Yunnan	HQ539761	HQ539864	HQ540072	HQ539971
A. zambiana	SYM	RET 343-10	Zambia, Copper Belt Province	HQ539762	HQ539865	HQ540073	HQ539972
Limacella glischra	FL	NY 00815491	Costa Rica, Puntarenas	HQ539763	HQ539866	HQ540074	HQ539973
L. affin. *illinaita*	FL	SFSU HDT 54917	United States, New Mexico	HQ539764	HQ539867		HQ539974
Pluteus cervinus	FL	F 88934	United States, Texas	HQ539765	HQ539868	HQ540075	HQ539975
P. petasatus	FL	NY 720956	United States, New York	HQ539766	HQ539869	HQ540076	HQ539976
Volvariella volvacea	FL	PSMCC, WC 439	China	HQ539767	HQ539870	HQ540077	HQ539977

*Specimens used in this study were either obtained via loans from herbaria or were collected from the wild. We obtained permits (when necessary, see below) for field collections or collected from public lands where permits were not required. We never collected from privately owned or protected areas. None of the species we collected are endangered or protected.

** See http://www.amanitaceae.org/ for full listing of all currently recognized symbiotic and free-living *Amanita* species

*** These specimens were collected by the first author from the wild. Some specimens with "BW_..." herbarium ID were collected from the following locations:

1) "BW_PH…." and "BW_HP" and "BW_HF" = Harvard Forest, Petersham, Massachusetts (with permit issued from the Harvard Forest);

2) "BW_STF…" = Stowe Town Forest, Stowe, Massachusetts (no permit required);

3) "BW_JLR" = Jake's Landing Road, Belleplain State Forest (with permit issued from the New Jersey State Park Service);

4) "BW_SH.." = Big Thicket National Preserve, Kountze, Texas (with permit issued by the National Parks Service);

and

5) "BW_RP.." = Rocky Point Natural Resources Conservation Area, Rocky Point, New York (with permit issued from the New York State Department of Conservation)
