# Supplementary material for: The Irreversible Loss of a Decomposition Pathway Marks the Single Origin of an Ectomycorrhizal Symbiosis
Source: PLoS One. 2012 Jul 18;7(7):e39597. doi: 10.1371/journal.pone.0039597 (PMC3399872; doi:10.1371/journal.pone.0039597)
Supplement: Table S2 — Primers used to amplify functional genes. (PDF) [file pone.0039597.s010.pdf]

Table S2: Primers used to amplify functional genes

	Primer name	Sequence	Targeted Protein Sequence
**Celulase gene screens**			
**Endoglucanase (*egl*)**	endoglucGH5f	ATYTTTGATGTYATGAAYGARCC	IFDVMNEP
	endoglucGH5r	CCACCAIGGWCCWGCIGC	WAGPWW
**Cellobiohydrolase I (*cbhI*)**	fungCBHIf*	ACCAAYTGCTAYACIRGYAA	TNCYTGN
	fungCBHIr	GCYTCCCAIATRTCCATC	EMDIWEA
**ß-glucosidase (*bg1*)**	inbetaglucGH3f	GGHGGWGGITCIGCIGCVCT	GGGSAAL
	inbetaglucGH3r	TGICGIGTCCAGTTRTCIATIAC	VIDNWTR
**Extracellular Aspartic Protease**			
	AsProtF1.2	GAYCAIGGCTCITCIGAYCTITGG	DTGSSDLW
	AsProtR2.3	GTGCCINWRTCGATIAYIGT	TIIDSGT
**RT-PCR primers**			
**ß-glucosidase (bg1)**			
Amanita thiersii	AthintBGf	GGCGGTGGGTCGGCGGCGCTGAAAG	GGGSAAL
	AallBGr	TGGCGGGTCCAGTTGTCGATGAC	VIDNWTR
Amanita cokeri	AcokintBGf	GGTGGTGGGTCGGCGGCCCTGACC	GGGSAAL
	AallBGr (see above)		
Amanita crenulata	AmuscintBGf	GGTGGAGGGTCGGCGGCGCTTAAAC	GGGSAAL
	AallBGr (see above)		
**elongation-factor 1 alpha (ef1α)**			
Amanita thiersii	AthefF	CAAGATGGACACTACTAAGTGGAG	RWTLLSG
	AallefR	CCGATACCACCGATCTTGTAGAC	RLQDRWYR
Amanita cokeri	AcokefF	CAAGATGGATACCACCAAATGGAG	RWIPPNG
	AallefR (see above)		
Amanita crenulata	AcrenefF	CAAGATGGATACCACTAAGTGGAG	RWIPLSG
	AallefR (see above)		

* Previously published primer pair (Edwards et al. 2008)
