# Supplementary material for: The Irreversible Loss of a Decomposition Pathway Marks the Single Origin of an Ectomycorrhizal Symbiosis
Source: PLoS One. 2012 Jul 18;7(7):e39597. doi: 10.1371/journal.pone.0039597 (PMC3399872; doi:10.1371/journal.pone.0039597)
Supplement: Table S3 — Cultures used in experimental assessment of saprotrophy. (PDF) [file pone.0039597.s011.pdf]

Table S3: Cultures used in experimental assessment of saprotrophy

Species Name	Strain ID	Source	GenBank Accession # of nucLSU Sequence
Amanita muscaria var guessowi (type 1)*	PS #283	Penn State Spawn Collection, #283 (from forest in United States, Pennsylvania)	HQ593121
Amanita muscaria var guessowi (type 2)	Koide BX008	Mushroom collected by Dr. Roger Koide, (from forest in United States, Pennsylvania)	HQ593120
Amanita crenulata	MM 9.01.06	Mushroom collected by authors, United States, Massachusetts	HQ593117
Amanita affin. *flavoconia*	PS 35	Penn State Spawn Collection, #35; (isolated in United States, Oregon)	HQ539663
Amanita affin. *brunnescens*	BW HF10C	Mushroom collected by authors, United States, Massachusetts	HQ539661
Amanita affin. *citrina*	BW PNC	Mushroom collected by authors, United States, New Jersey	HQ539662
Amanita cinereoconia	BW_PSF	Mushroom collected by authors, United States, New Hampshire	HQ593118
Amanita polypyramis	BW_CC	Mushroom collected by authors, United States, Massachusetts	HQ593122
Amanta cokeri	BW_STF 090506-19	Mushroom collected by authors, United States, Massachusetts	HQ593113
Amanita inopinata	Kibby_2008	Mushroom collected by authors, United Kingdom, Suffolk	HQ593116
Amanita thiersii	SKay4041_het	Mushroom provided to authors by Sherry Kay, United States, Kansas	HQ593114
Amanita manicata	Hemmes_2008	Mushroom collected by authors, United States, Hawaii	HQ593115
Volvariella bombycina	PS# WC 427	Penn State Spawn Collection, WC 427 (isolated from wood, United States, Michigan)	HQ593119
Volvariella volvacea	PS# WC 439	Penn State Spawn Collection, WC 439 (isolated in China)	HQ539767

*The two *Amanita muscaria* var. *guessowii* strains represent two genetically distinct types based on SNPs identified in the ITS sequences of these strains. They are placed in the I/A NE US clade of the *Amanita muscaria* species complex identified by Geml J, Tulloss RE, Laursen GA, Sazanova NA, Taylor DL. 2008. Evidence for strong inter- and intracontinental phylogeographic structure in *Amanita muscaria*, a wind-dispersed ectomycorrhizal basidiomycete. Mol Phylogenet Evol 48(2):694-701.
